# On the Nerves of the Uterus

**Published:** 1846-10-01

**Authors:** 


I. On the Nerves of the Uterus. By *Thomas Snow Beck*, Esq. Surgeon. Communicated by Sir *Benjamin C. Brodie*, Bart. F.R.S. Philosophical Transactions, 1846. Part 2.

II. Supplement to a Paper "On the Nervous Ganglia of the Uterus." By *Robert Lee*, M.D. Fellow of the Royal College of Physicians, London. Philosophical Transactions: 1846. Part 2.

Many of our readers are aware that a controversy has arisen respecting the award of one of the medals of the Royal Society to Mr. Beck, for his investigation of the nerves of the Uterus. Although our principal object in the present article, will be to consider the subject matter of the papers, the titles of which are given above, it would be a dereliction of our duty as journalists, were we to pass by unnoticed, the complaints of injustice which have been brought forward against the Council and Physiological Committee of the Royal Society in reference to this transaction. The facts of the case, so far as an impartial consideration of the involved assertions and contradictions of the numerous parties implicated will enable us to form a judgment, are essentially as follows: a paper "On the Nerves of the Uterus," by Mr. Beck was presented, but not read, the title alone having been announced, to the Royal Society at their meeting on June 19, 1845. On the same evening a paper from Dr. R. Lee, entitled "Supplement to a Paper on the Nervous Ganglia of the Uterus," was presented and read. Mr. Beck's paper was referred by the Committee of Physiology to Drs. Todd and Sharpey, for the purpose of deciding the question of its being printed. On the 28th of October last, the Committee met to receive the report of the two referees, when, at the commencement of the meeting, Dr. Todd only was present and prepared with his report; Dr. Sharpey subsequently arrived, but unprepared with any report. At this meeting Mr. Lawrence presided, and, after the routine business had been transacted, Dr. Roget, one of the Secretaries, having by mistake stated that the Royal Medal for the year was not to be awarded in the physiological section, Mr. Lawrence vacated the chair and left the room. Subsequently to the departure of the chairman, it was discovered by Dr. Roget that he had committed an error; that a medal was to be awarded for the best paper on physiology during the preceding triennial cycle; and that it was requisite the selection and recommendation should be made on that day. On this, the Committee was re-formed, Dr. Todd being now in the chair; a list of all the physiological papers that had been read during the last three years, fifteen in number, was made by Mr. Bell, the Secretary of the Physiological Committee; and, ultimately, Mr. Beck's paper was recommended to the Council for the award of the Royal Medal.

Such is a very brief, but we believe correct, statement of a proceeding which has excited a large amount of animadversion and comment; and we do not for a moment hesitate in expressing our unbiassed opinion, that there is much ground for rebuke in the whole transaction. In the first place, we entirely agree with our contemporary, the Editor of the Lancet, that the award was irregular and of questionable legality, inasmuch as it was made in direct contravention of the following regulation, approved by the Queen and affixed yearly to the Philosophical Transactions: "that the Royal Medals be given for such papers only as have been presented to the Royal Society, and inserted in their Transactions." The paper of Mr. Beck, which was never read before the Society, has only now been published nine months after the award. In the second place, it appears to us that the Physiological Committee acted, to say the least of it, with impropriety, by adopting any resolution in the absence of a document deemed by themselves requisite to guide their judgment, the written report, namely, of Dr. Sharpey. Further, the proceedings of the Committee were allowed by the responsible officers of the Society to be conducted in a manner so slovenly and hasty, that the members, when assembled to perform the most important of all their functions, were in total ignorance of the business which was to come before them. So long as irregularities like these are permitted, there can be no security against partiality and injustice; and though in the present case we are convinced, knowing the upright and honourable character of those present, that no intentional unfairness influenced the decision, still it cannot be admitted that, in such a hurried scrutiny, fair and even-handed justice swayed the award. Time may shew, as we think it will, that although Mr. Beck's paper does not present that amount of original observation or discovery to which alone, as a rule, these Royal Medals should be awarded; yet, that it contains so many important facts, and is calculated to obviate so much uncertainty respecting the pelvic nerves, as to justify a deviation from what ought to be the fixed rule of procedure. But being an exception, and bearing upon a question which is still sub judice, the arbitrators must be content to bear whatever blame attaches to hasty and ill-considered measures, in a matter above all others demanding delicacy and deliberation.

It is well known that Dr. Robert Lee, in two papers published in the Philosophical Transactions for 1841 and 1842, described and depicted the nerves, and especially the ganglia situated on the neck of the uterus, as being remarkably enlarged during pregnancy. The account thus given rested on some most careful dissections of the gravid uterus made by Dr. Lee; and which, as that gentleman states in his appeal to the President and Council of the Royal Society against the award made to Mr. Beck, have been examined by more than fifty distinguished English and foreign anatomists, who have borne testimony in writing to the truth of the published statements.* In the volume of the Philosophical Transactions just published, Dr. Lee has given the results of another dissection, which, he affirms, corroborates his former account.

"In a gravid uterus at the full period I have recently, and with still more care, traced the great sympathetic and spinal nerves into the two hypogastric ganglia, and from thence over both sides of the uterus to the fundus. A lens which magnified six diameters was employed in this dissection, which enabled me with unerring certainty to distinguish and to separate the nervous filaments from the fine cellular membrane by which they are so closely surrounded, and from all the other

---

* See Letter from Dr. Lee to the President and Members of the Council of the Royal Society of London.—*Lancet*, May 9, 1846, p. 527.

contiguous structures. In this minute dissection, many of the details of the nervous system of the uterus are more perfectly shown than in any previous dissection made by me, and they confirm, in the most complete manner, the accuracy of all that is contained in my previous communications on this subject to the Royal Society. To this preparation I can now appeal, as affording a perfect demonstration of the truth of all my statements respecting the ganglia and other nervous structures of the uterus."—*Phil. Trans.* 1846, Part 2, p. 211.

In the Abstract of the "Proceedings of the Royal Society," of April 2, 1846, an outline of "Further Researches on the Nervous System of the Uterus" is printed; but the only additional fact here brought forward by Dr. Lee is that Mr. Dalrymple, whose accuracy as an observer is well known, has made a "microscopic examination of the uterine nerves in preparations furnished by the author, which tends to corroborate his views."—*L. c.* p. 610.

It thus appears that Dr. Lee abides by his opinion, that, during pregnancy, the nervous system of the uterus is enlarged, and to such an extent that one of the many ganglionic masses, the utero-cervical ganglion, exceeds in size the semi-lunar ganglia of the great sympathetic. The researches of Mr. Beck, now for the first time published, have led that gentleman to conclusions of an exactly opposite character; for he contends that "the nerves undergo no change of size during pregnancy, nor of position, except such as results from the enlargement of the organ over which they are distributed."—(*Phil. Trans.* 1846, Part 2, p. 214.) The dissections supporting this assertion were made on one uterus only, being that of a woman, aged thirty-five years, who had died from hæmorrhage immediately after delivery. The original object of Mr. Beck in making the examination, and the circumstances attending it are thus stated:—"With the view of confirming the researches of Dr. Lee, I commenced the dissection of the nerves of the gravid uterus, but found so many points at variance with his published statements, and the nerves so small, considering the size of the organ, that it appeared very doubtful whether or not they had increased in size during pregnancy; but in order to arrive at a correct conclusion on this point, a comparison of the nerves of the unimpregnated with those of the gravid uterus was indispensable; and in the following year I commenced a dissection of the nerves of the unimpregnated uterus and of the neighbouring organs."—*L. c.* p. 213.

Although the investigation was directed more especially to the uterine nerves, the author was led, in the elaborate dissection which he prosecuted with a rare perseverance for a period of two years, to several other points of great interest relating to the anatomical connexions and structure of the great sympathetic nerve. It is to these last-named parts that we would, in the first instance, direct the attention of our readers.

It has always been deemed a point of physiological importance, to determine the exact nature of the communications existing between the spinal nerves and the great sympathetic; and for this obvious reason, that it is evidently through their medium that all the impressions passing reciprocally between the cerebro-spinal system and the ganglionic system, must traverse. Physiological experiment, equally with pathological observation, demonstrates that such modes of transmission do exist: for instance, in a decapitated kitten, irritation of the spinal cord causes an acceleration of the heart's action; and again, showing a passage in the opposite direction, irritation of the mucous surface of the intestine, often determines convulsion in the voluntary muscles. Mr. Beck, carrying his investigations further than those of other observers, has proved, by noting the microscopic characters of the fibres, that, in the branches of communication between the sympathetic and spinal nerves, there are contained both gray and white filaments; and he has likewise traced these to their ultimate distribution.

The following is the author's account of this intricate piece of anatomy: "The branches of communication between the spinal nerves and the thoracic ganglia consist of two distinct portions, marked by difference in colour, consistency and distribution. One portion, the smaller of the two, is soft, semitransparent, and of a light brown colour. It leaves the posterior border of the thoracic ganglion, and joins the spinal nerve about one-eighth of an inch anterior to the giving off of the white portion; a gray communicating cord also passes between the thoracic ganglia. This ganglionic mass, when first exposed in the recent dissection, was of a deep red-brown colour, which quickly faded to a light brown by the action of the water in which it was examined. The other portion, about one-half larger than the former, is firm, of an opake pearly-white colour; it passes to the anterior surface of the ganglion, and divides into two portions. One of these portions joins with a similar part, sent down from the ganglion next above, and then goes out in a curving direction on the side of the dorsal vertebræ to contribute in the formation of the splanchnic nerve. The other half of this branch of the intercostal nerve turns down, constituting a part of the intercommunicating cord between the ganglia, to the ganglion next below, and there joining with a similar portion from the intercostal nerve on a level with this ganglion, passes forwards to join the other branches which form the splanchnic nerve. Each branch sent to the splanchnic nerve contains branches from at least two intercostal nerves, viz. from the nerve on the level with its apparent origin, and from the nerve next above it. This arrangement of the nervous fibres is very apparent in the recent dissection, from the marked distinction which exists in colour and consistency between the gray and white portions of the so-called trunk of the sympathetic."—(*L. c.* p. 214). It is, however, important to state, that the branches forming the splanchnic nerve are not exclusively formed of the white fibres of the spinal nerves, but "are partly composed of gelatinous nervous fibres derived from the thoracic sympathetic." Thus the splanchnic nerve is composed of cerebro-spinal (tubular) fibres and ganglionic (gelatinous) fibres; and "these two kinds of fibres, although associated together, are yet as distinct as the motor and sensitive tubules of the cerebro-spinal nerves, after they are mixed together."

The relations of the gray or gelatinous fibres, passing in the communicating branches, are so interesting that it is desirable to give the author's account of them. These "gray roots of the sympathetic," as they are usually called by anatomists, are given off from the thoracic ganglia; some of these gelatinous fibres "join the intercostal nerve, and, becoming associated with other gelatinous fibres which arise from the ganglion on the sensitive root of the spinal nerve, go with the intercostal nerve in its distribution. The ganglia on the sensitive root of the spinal nerves become thus associated with the ganglia of the sympathetic, whose office, anatomically considered, is to give origin to the gelatinous fibre. A few gelatinous fibres are found in both the motor and sensitive roots of the spinal nerves, but the quantity is so small, when compared with the quantity found in other parts, as to preclude the idea that the sympathetic arises from them. Most probably these fibres are distributed to the vessels of the cord; as gelatinous fibres, which come from the gray branch of the thoracic ganglion, pass along the motor root in the direction of the spinal cord, and gelatinous fibres from the same source, also enter the ganglion on the sensitive root: both these sets of fibres evidently pass from the thoracic ganglion towards the spinal cord."—(*L. c.* p. 215.) Mr. Beck sums up this division of his inquiry by observing, " we have thus two distinct and separate systems of nerves, one composed of gelatinous nervous fibres which have their origin at the different ganglia; the other composed of tubular nervous fibres, which arise at the brain and spinal cord."

He further has confirmed the opinion generally entertained, that the so-called gelatinous fibres are in reality true nervous fibres, by ascertaining the facts that many small nerves are entirely composed of them, and that the ultimate distribution of many of them is to the arteries. The sole anatomical use of the ganglia is conceived to be " to give origin to the gelatinous fibres, to which end they are distributed irregularly over the body, in those situations where a supply of the gelatinous fibre is required. The true sympathetic system appears to be a system of gelatinous nervous fibres, which are distributed everywhere over the body, and which preside over the organic functions. It appears to exist independently of the brain and spinal cord, although the tubular fibres which come from the brain and spinal cord are associated with the gelatinous fibres in the larger branches of the sympathetic."—(*L. c.* p. 219). All these are important details, confirming in many essential points the views arrived at by other modes of research; they also afford another illustration of Bell's one fundamental principle—the individuality of the primitive nervous fibres; and, by showing that the great sympathetic, with all its intricate plexuses obeys the same disposition as that established in the cerebro-spinal axis, prove that, throughout the entire nervous system, there is but one law of conduction.

We now come to that part of these researches which constitutes the main object of the whole enquiry, the anatomy of the nerves of the uterus. The clue to the cause of the discrepancy between these dissections of Mr. Beck and those of Dr. Lee, is indicated by the following note, appended by the former gentleman to his paper, and dated May, 1846. " In the progress of my dissections, I found that the structure considered by Dr. Lee as 'the hypogastric or utero-cervical ganglion' was a mass of dense fibro-cellular tissue enveloping several small ganglia and a nervous plexus, formed at the junction of the lateral hypogastric plexus with branches from the sacral nerves; that the 'vesical ganglia' were also a mass of fibro-cellular tissue, in about the centre of which were situated small ganglia; and that the remaining structures described as ganglia and plexuses were not nervous structures."—(*L. c.* p. 213.) What these non-nervous structures really consist of, according to Mr. Beck's views, may be understood by one of the references to the plates: " R. R. Portions of the superficial layer of muscular fibres (of the uterus) which have been dissected out by Dr. Robert Lee, and described by him as nervous ganglia and plexuses. These muscular fibres adhere to the under surface of the peritoneum."—*L. c.* p. 234.

Our limits will not permit us to follow the intricate plexuses of nerves situated in the pelvis, so minutely followed and traced out by Mr. Beck; it would, however, be unjust towards that gentleman, if we did not state that his account differs in some respects from that of former writers, and that he has more successfully determined the source and disposition of those nerves than has hitherto been accomplished. One or two points are all that we can notice. The author has ascertained that there is an important difference in the constitution of the nerves supplying the bladder and vagina, organs endowed with a high degree of sensation, especially the vagina, and also with voluntary muscular power, when contrasted with those proceeding to the uterus, rectum and intestines; in the former case, the nerves contain a much larger amount of tubular (cerebro-spinal) fibres, than in the latter. The precise arrangement, extent, and relations of the the lateral hypogastric plexuses, springing from the inferior aortic plexus of the sympathetic, of the branches of the sacral plexus, and of the pelvic plexus formed by the union of the two former, are most satisfactorily demonstrated. Among other points that have been ascertained, it appears that no branches of the sacral nerves are sent to the neck of the uterus, as some have imagined upon physiological grounds to be the case.

The author thus speaks of the much controverted question, respecting the size of the nerves in the unimpregnated and impregnated uterus:—

"The Size of the Nerves.—The size of the nerves in both these dissections (that is of the unimpregnated and impregnated uterus) is essentially the same, and when the nerves are carefully compared, no doubt is left that the nerves of the gravid uterus have undergone no change in size; nor any change in position, except that consequent upon the development of the organ. Yet at first sight the nerves of the unimpregnated uterus appear even larger than those of the gravid uterus; this, however, is due to the manner in which they are arranged. In the unimpregnated state of the uterus, the nerves of the lower portion of the hypogastric and pelvic plexuses, are crowded and packed together so as to occupy a much smaller space than they do in the gravid state of the uterus. The consequence of this is, that the nerves of the former present a wavy arrangement, which gives them the appearance of greater thickness and plumpness."—*L. c.* p. 221.

We may remark that we have observed in the œsophagus of the Boa Constrictor, a part subject to occasional distension, a wavy disposition of the nerves exactly similar to that above described by Mr. Beck, and which would indicate that, in pregnancy, the uterine nerves, we say nothing of the ganglia, are unfolded rather than enlarged.

As to the number of nerves belonging to the uterus, it is, according to the author, remarkably small; a few isolated branches are traced, "which pass on as distinct fine cords, dividing and subdividing, but not uniting with each other." The lower part of the organ is supplied by the lateral hypogastric plexus; the middle and upper part has a distinct branch from the inferior aortic plexus; whilst the fundus receives a branch from the nerve which, coming off from the renal plexus, accompanies the spermatic artery. Mr. Beck also speaks of another set of nerves, minute in size, assuming a plexiform arrangement around the vessels, and having the distinctive character of forming here and there minute ganglia.

With respect to the vessels of the uterus, the author is inclined to think that they do not diminish in size after parturition, but are only contracted in their cavity, ready to be again stretched out upon a larger portion of blood being sent to the organ.

In consequence of the great interest that the controversy respecting the merits of Dr. Lee's and Mr. Beck's papers has excited, we have deemed it desirable to furnish our readers with all the details bearing upon the question; and the nature of the inquiry is the best apology for the many anatomical facts contained in the present article. As to the merits of the case, it seems, according to our judgment, that further examinations are required for finally arriving at the truth; and it is especially desirable that the disputed textures—we allude particularly to the large masses which Dr. Lee contends are true ganglia, *should be examined microscopically in a perfectly recent condition;* and this is an examination, which that gentleman should have made forthwith. Under such circumstances the existence or non-existence of the nerve-corpuscles, the only certain test of ganglionic substance, would definitely determine the question, and no other kind of proof will be deemed satisfactory by impartial persons. It may not be superfluous to add that, having examined the dissections both of Dr. Lee and Mr. Beck, we are inclined to receive those of the latter gentleman as demonstrating the real structure, although we were among those who formerly admitted the accuracy of Dr. Lee's preparations. Nothing can exceed the beauty of Mr. Beck's specimens; and the plates illustrative of them, are equally to be admired.

As we have had occasion in the first part of this article, to refer in condemnatory language to the proceedings connected with the award of the Royal Medal to Mr. Beck, it is proper to state that our remarks were aimed at what we deem to be the vicious system permitted to exist in a national institution, and were not intended to depreciate the value of these very important researches, which merit, as they will doubtless receive, the highest praise.

